# Peer support to decrease diabetes-related distress in patients with type 2 diabetes mellitus: design of a randomised controlled trial

**DOI:** 10.1186/1472-6823-14-21

**Published:** 2014-03-04

**Authors:** Lianne de Vries, Amber AWA van der Heijden, Esther van 't Riet, Caroline A Baan, Piet J Kostense, Mieke Rijken, Guy EHM Rutten, Giel Nijpels

**Affiliations:** 1 Department of General Practice and Elderly Care Medicine, VU University Medical Center, Amsterdam, The Netherlands; 2 Department of Epidemiology and Biostatistics, VU University Medical Center, Amsterdam, The Netherlands; 3 EMGO + Institute for Health and Care Research, VU University Medical Center, Amsterdam, The Netherlands; 4 RIVM, National Institute for Public Health and the Environment, Bilthoven, The Netherlands; 5 NIVEL, Netherlands Institute for Health Services Research, Utrecht, The Netherlands; 6 Julius Center for Health Sciences and Primary Care, University Medical Center Utrecht, Utrecht, The Netherlands

**Keywords:** Peer support, Diabetes mellitus type 2, Distress, Randomised controlled trial

## Abstract

**Background:**

Many type 2 diabetes mellitus patients face difficulties self-managing their illness, which can lead to high levels of diabetes-related distress. Diabetes distress may be decreased by peer support, as peers understand and have dealt with similar problems, and can help motivate each other. A recent systematic review concluded that evidence of benefits of peer support in patients with type 2 diabetes mellitus is too inconsistent due to weak theoretical foundation of the interventions. This study describes the design of a trial evaluating the effectiveness of a group-based, peer support programme with a strong theoretical foundation on diabetes-related distress in type 2 diabetes patients.

**Methods:**

This is a parallel group randomised controlled trial of a six session group-based peer support intervention, delivered by peer leaders and group psychotherapists, compared with one educational meeting on diabetes. At least 152 patients with a type 2 diabetes duration of three years or more and between 50 and 70 years of age, recruited via their general practitioner, will be randomised to receive the peer support intervention or one educational meeting. The intervention is developed in line with three key stages of research development of the Medical Research Council framework. The primary outcome measure for this study is diabetes-related distress. Secondary outcomes include self-management behaviour, well-being and health-related quality of life. Perceived social support is a process measure. Outcomes will be measured one month before, and 6, and 12 months after the intervention by means of self-reported questionnaires. Analysis will be on an intention-to-treat basis.

**Discussion:**

This article contains a description of the design of a study that will investigate the effect of a group-based, peer support intervention on diabetes-related distress in type 2 diabetes patients. The intervention was developed in recognition of the limited evidence, and the importance of a theoretical foundation and its implementation. Findings will contribute to knowledge in the field of peer support and patient-important outcomes in type 2 diabetes patients.

**Trial registration:**

Dutch Trial Registry: NTR3474

## Background

Type 2 diabetes mellitus (T2DM) is a chronic disease that is becoming more prevalent across the world [1] and is placing increasing demands on healthcare systems [2]. There has been a growing focus on the involvement of patients in chronic disease management by having patients make informed choices with respect to lifestyle changes related to exercise, diet, medication and self-monitoring. However, many patients face difficulties making these lifestyle changes, which can lead to high levels of diabetes-related distress [3]. Diabetes-related distress is often associated with difficulties in coping with a daily regimen and worries about developing late complications [4]. This type of distress can manifest itself in several ways: feeling that one is not capable of managing diabetes well enough; feeling overwhelmed by self-care regimens; and feeling that others do not understand the difficulty of managing diabetes [5]. Psychological distress is not only burdensome in and of itself, but can also impede patient self-care, thereby compromising glycaemic control [4,6].

Diabetes-related distress may be decreased by peer support interventions. Peer support has been defined as the provision of support by an individual with experiential knowledge based on shared life experiences [7]. It is a promising approach as it harnesses the ability of patients with T2DM to support each other in managing their day-to-day lives. Peers have dealt with many similar problems and understand a situation in a way that family members and friends likely cannot, as they often lack knowledge and the experience of dealing with diabetes in daily life [8]. Peers can support each other to stay motivated and help deal with the stress chronic disease often brings [9].

A recent systematic review of the effect of peer support on diabetes outcomes in adults concluded that peer support appears to benefit some patients with T2DM, but that the evidence provided by fourteen randomised controlled trials (RCTs) is too inconsistent to draw firm conclusions [10]. Only seven of the fourteen RCTs took health distress or depression into account, which renders the evidence concerning diabetes-related distress, the primary outcome of this study, limited. Dale et al. state that there may be considerable scope for increasing the effectiveness of peer support intervention by strengthening its theoretical foundation and linking this to the processes involved in all aspects of its implementation [10].

In order to strengthen the theoretical foundation and the design of intervention studies, the current study proposal incorporates three key stages of research development in line with the Medical Research Council (MRC) framework: establishing a theoretical basis, feasibility testing, and pilot testing the intervention [11]. This has led to what makes our peer support intervention unique, namely voluntary laypeople with T2DM and group psychotherapists together leading the peer support groups. While laypeople serve as positive role models for participants and can share similar first-hand experiences of living with T2DM, group psychotherapists are highly skilled at managing group discussions and dynamics. We hypothesise that the collaboration between laypeople–or peer leaders–and group psychotherapists and the combination of their skills secures the delivery of the actual intervention.

This article describes the study design and methods of a RCT of a group-based, peer support programme for patients with T2DM. The intervention was developed in recognition of the limited evidence, the importance of a theoretical foundation and its implementation, and the need to support peer leaders in securing the key element of the intervention. The aim of the study is to determine the effectiveness of a group-based, peer support programme on diabetes-related distress. We hypothesise that participation in a group-based, peer support programme decreases diabetes-related distress leading to an improvement in secondary outcomes such as self-management behaviour, well-being, and health-related quality of life.

## Methods

### Design

This study is a RCT approved by the Medical Ethics Committee of the VU University Medical Center in Amsterdam, the Netherlands.

### Patients and practices

Participants for this study will be recruited from 130 general practices in the northwestern, middle and southern parts of the Netherlands. At each practice site a member of the research team will search the registers for individuals with T2DM who meet the following inclusion criteria:

– treated for T2DM in a primary care setting at one of the three study sites

– between 50-70 years of age

– a diabetes duration of at least three years

To increase the likelihood of an effective peer support intervention, we will recruit patients from the same age group and the same phase of illness. Evidence suggest that peers closer in age have an increased likelihood of providing effective peer support and peer support is especially beneficial when patients are tackling challenging new developments in their disease such as complications [12,13]. Patients who do not speak or understand the Dutch language; and those with severe accompanying disorders (e.g. mentally ill; severe learning difficulties) will be excluded.

All potential participants will receive an invitation to participate. Patients will be provided with written information about the study, invited to give written consent. The study team will not have access to the personal data of the patients. After inclusion, patients will be randomly assigned to the intervention or the control arm. This means usual care plus participation in a group-based peer support programme consisting of six sessions or usual care plus attendance of one educational meeting on T2DM respectively.

Randomisation will be carried out electronically by a researcher who will have no day-to-day involvement with the trial’s administration. The randomisation status will remain unknown to the research team until all participants are recruited and the peer group sessions require co-ordination. The randomisation status will be kept hidden from the analyst until the analysis is essentially complete.

### Intervention development

In line with the MRC framework for development and evaluation of RCTs for complex interventions to improve health [11], this study has integrated three phases of intervention development.

1) Preclinical phase

In the first phase, a theoretical basis was established on the basis of relevant literature. The theoretical background of this peer support intervention lies within the social support model [7,14]. This model hypothesises that individuals who experience support are likely to have a better quality of life, fewer negative feelings and are thought to take better care of themselves. Three elements appear repeatedly in the descriptions of peer support interventions: emotional, informational, and appraisal support [7]. Emotional support concerns the possibility of discussing personal difficulties with another person. It is associated with sharing life experiences and involves the exchange of empathy, trust and caring [15]. Informational support is the exchange of advice, suggestions and information relevant to problem solving [16]. Appraisal support involves the exchange of information that is useful for self-evaluation purposes: constructive feedback, affirmation and social comparison [7]. Informational support and appraisal support are often combined into one social support domain [17].

During peer support interventions patients not only receive informational, appraisal and emotional support, but patients also get the chance to support others. Therefore, peer support can combine the health benefits of both receiving and providing support. Evidence exists that suggests that providing support may result in health benefits comparable to–or even greater than–receiving support [18]. Individuals who provide social support experience less depression, a heightened sense of self-efficacy and self-esteem, improved quality of life and health behaviours, and decreased mortality risk, even after adjusting for baseline health status and socioeconomic status [18].

**Figure 1:**
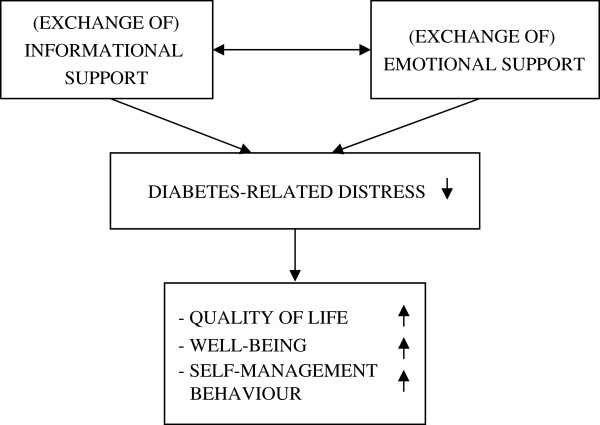
Theoretical model: hypothesised effects of peer support.

Figure 1 shows two elements of peer support and their hypothesised effects. Our group-based, peer support intervention is expected to lead to a decrease in diabetes-related distress. This decrease may then result in improved health-related quality of life, well-being, and self-management behaviour.

2) Modelling phase

In the second phase we explored the acceptability and feasibility of the proposed intervention through qualitative research. Two focus groups were conducted with patients with T2DM (n = 10), experienced in leading courses on diabetes, including peer support, recruited via the Dutch Diabetes Association [19]. Interviews lasted two hours and were recorded and transcribed. Data were coded in ATLAS.ti [20] for core codes of interest to the study (e.g. characteristics of peer leaders, design of the intervention, and delivery of peer support). Peer support was felt to be a suitable way to learn from the advice and experience of others and to feel better equipped to manage diabetes in daily life. However, participants felt that peer support is a complex intervention and that it might not be enough to simply train the laypeople leading the groups. Peer leaders might need the support of a professional experienced in group dynamics and the encouragement of sharing of emotions. This has led to the idea of shared leadership of the peer groups consisting of a layperson and a group psychotherapist. In addition, the focus groups resulted in a first draft of the peer group intervention. Participants believed six peer group meetings over six months would be satisfactory and that meetings should last no longer than two hours.

This first draft of the intervention was introduced to potential participants. A total of 65 T2DM patients from one general practice were invited to attend, and 25 T2DM actually attended an education session on diabetes during which the peer group intervention was presented. Attendees associated peer groups with problem support groups and expressed their need for more knowledge on their disease. To overcome the problem of the negative image of peer support and respond to the need for knowledge, we decided to integrate our peer support intervention with a course on T2DM. Peer support and promoting exchange and discussion among participants remains the vital element of the intervention.

3) Pilot phase

The third phase was to undertake a pilot test of the feasibility of the intervention. The pilot consisted of one fully-trained peer leader and a trained group psychotherapist delivering one peer group meeting together for six patients with T2DM from one general practice in one of the three research regions. A total of 27 patients with T2DM received an invitation letter to attend the meeting. Evaluation afterwards revealed that patients were satisfied with the mixture of information on diabetes and the exchange of experiences with other patients and the peer leader. Positive outcomes of the pilot were also reported by the peer leader and group psychotherapist. During the pilot session, the two leaders mostly observed (the exchange of) informational support. Emotional support is more likely to occur in subsequent sessions when participants get to know each other and feel safe enough to share emotions.

### Intervention

Patients in the intervention arm of the trial take part in a peer support programme consisting of a two-hour session once a month for six months. Peer leaders teach the programme in an interactive manner designed to enhance participants’ confidence in their ability to execute specific self-care tasks (self-efficacy). The goal is not to just provide disease-specific content, but rather to use interactive exercises to build self-efficacy in order to help participants to better cope their condition. A vital element is promoting exchange and discussion among participants on topics such as medication adherence, diet and exercise, communication with health care providers, and worries about possible complications. In doing so, situations are created in which the exchange of informational support and emotional support takes place. This is part of the role of the group psychotherapist. To facilitate participant attendance, the programmes will be held in easily accessible community centres in the participants’ own neighbourhood during the afternoon and evenings. Participants are encouraged to meet and/or keep in touch and support each other after the six peer group meetings.

### Peer leaders and group psychotherapists

The peer leader leads the group to share experiences and give advice, and to encourage each other to be proactive regarding his or her diabetes care. All eight recruited peer leaders were diagnosed with diabetes over ten years ago. They have worked for the Dutch Diabetes Association and have had at least five years of experience working as a diabetes educator. Following recruitment, the peer leaders completed one day of training. The training included an introduction to the study; the role of the peer leader and its limitations (i.e. not being a medical doctor); communication and role-playing to practice handling difficult situations. In addition to the training, peer leaders are supported during the intervention period by means of monthly phone calls after each session with a member of the research team.

During the group meetings, the peer leaders are supported by a group psychotherapist. The peer leader and the group psychotherapist form a team. The role of the group psychotherapist is to manage group dynamics and help the peer leader to stimulate interaction between the participants. The four group psychotherapists were recruited through the network of the research team. Like the peer leaders, the group psychotherapists received training on their role in the intervention. They were also instructed to fill out a form to evaluate each peer group session (i.e. which forms of peer support were observed).

### Control group

Participants allocated to the control arm continue to receive their usual diabetes care–as do participants in the intervention group–and receive an invitation to attend just one educational meeting in their neighbourhood. During this meeting, a professor in general practice and diabetes care will instruct participants on nutrition, exercise and medication. By offering the possibility of attending a meeting and receiving information, both the intervention and the control group receive some form of attention.

### Data collection and outcome measures

Outcome measures will be assessed at baseline (T0) and directly after the intervention at six months (T1) and at twelve months (T2) by means of self-reported questionnaires. The primary outcome is diabetes-related distress. Health-related quality of life, well-being, and self-management behaviour will be reported as secondary outcomes. Perceived social support will be considered a process measure to evaluate whether the intervention will in fact lead to an increase in the providing and receiving of social support. The questionnaire will also include questions regarding patients’ sociodemographic and illness characteristics, for example sex, age, material status, ethnic origin, education, treatment of T2DM and complications.

### Primary outcome measure

– Diabetes-related distress is measured using the validated Problem Areas In Diabetes questionnaire (PAID) [4]. The PAID is a self-report questionnaire that consists of 20 statements identified as common negative emotions related to living with diabetes. Each item is rated on a 5-point Likert scale, ranging from 1 (“not a problem”) to 5 (“a serious problem”). Internal consistency of the Dutch PAID is high and stable across sex and type of diabetes (0.93-0.95). Test–retest reliability is high with a Pearson’s correlation of 0.83 [21]. The pattern of findings reported by Welch et al. [21] provide strong support of the responsiveness of the PAID.

### Secondary outcome measures

– Health-related quality of life is assessed using the well-validated EQ-5D. EQ-5D consists of the EQ-5D descriptive system and the EQ visual analogue scale. The EQ-5D descriptive system comprises the following five dimensions: mobility, self-care, usual activities, pain/discomfort and anxiety/depression [22].

– General psychological well-being will be assessed using the validated Dutch version of the self-report WHO-Five Well-being Index [23]. The five item questionnaire covers positive mood (good spirits, relaxation), vitality (being active and waking up fresh and rested), and general interest (being interested in things) [24].

– Self-management behaviour of the patient is assessed using the validated Dutch version of Glasgow’s Diabetes Self-Care Activities Measure. The DSCA is a brief self-report questionnaire consisting of eleven items assessing general diet, specific diet, exercise, blood glucose testing, foot care and smoking [25].

– Social support received will be evaluated using the Diabetes Support Scale [26]. The DSS is a brief self-report questionnaire consisting of eight items assessing the extent to which patients (feel they) receive informational and emotional support. To be able to measure the support they provide as well, four self-developed items will be added to this questionnaire. We will conduct a validation study of this new instrument called the Diabetes Receiving and Providing Support Scale (DRAPSS).

### Sample size

The sample size per group is calculated as ((Z_(1 - *α*/2)_ + Z_(1 - *β*)_)^2^ × 2*σ*^2^)/*δ*^2^, where δ is the minimal important difference to be shown between the means of both groups (intervention vs. control), σ is the standard deviation, α is the type I error rate, and β is the type II error rate. α is set at 0.05 (double-sided, Z_α_ =1.96) and power at 80% (*β* = 0.20, Z_*β*_ = 0.84). The proposed trial is designed to detect a clinically relevant change in diabetes-related distress. No consensus exists about minimal important differences (MID) of distress measured with the PAID. Therefore, we set the MID (δ) at half of a standard deviation. This is a well-known solution when scores have no direct interpretation and no clinical results exist to determine a relevant percentage. In diabetes patients, the standard deviation (SD) of PAID (scores transformed to 0-100) was 20 points [4]. This leads to a sample size of 63 per group required to find a difference of 10 points (0,5 SD). Allowing for an attrition rate of 20% from initial recruitment, 76 subjects in each arm are required (i.e. 152 participants in total).

### Analyses

On the basis of an intention-to-treat analysis, differences in outcome measures between the intervention group and control group are calculated with 95% confidence intervals. In addition, per protocol analyses that only include participants that attended three or more group sessions will be performed. In the unlikely case that, in spite of randomisation, an important prognostic factor is unequally distributed over groups, the analysis will be adjusted. Analysis of (co)variance, linear and logistic regression will be used to determine the effect of the intervention on each of the outcome measurements.

## Discussion

Peers support is a way for patients to stay motivated and help each other to deal with the stress T2DM often brings. This article contains a description of the design of a study that will investigate the effect of a group-based, peer support intervention on diabetes distress in patients with T2DM.

As previously stated, there is limited and inconsistent evidence on the effect of peer support on diabetes outcomes in adults with T2DM [10]. This trial will deliver important additional insight into the effects of peer support on patient-important outcomes like diabetes distress. To increase the effectiveness of our peer support intervention, the design is based on the MRC Framework [11] and on the social support model [7]. Both frameworks will contribute to the interpretation of the final results and will facilitate the reproducibility of our unique intervention, led by both voluntary laypersons with T2DM and group psychotherapists.

The actual delivery of the intervention may highlight some limitations, however. Concerns may arise regarding the dependence on (and the difference in) the knowledge and skills of the peer leaders and group psychotherapists. Training ensures that peer leaders and group psychotherapists are familiar with the dimensions of peer support and are capable of judging whether or not all dimensions are dealt with during the group sessions. Another concern may be the self-selection of participants as a potential threat to external validity. Because little is known about T2DM patients who take part in (peer) group interventions, we plan to conduct a non-response analysis and describe the reach of our programme.

To conclude, the present study will evaluate the effects of a group-based, peer support intervention led by a peer leader and group psychotherapist on diabetes-related distress, well-being and health-related quality of life, self-management behaviour, and perceived social support in patients with T2DM. Our findings will contribute to knowledge in the field of peer support and T2DM.

## Abbreviations

T2DM: Type 2 diabetes mellitus; RCT: Randomised controlled trial; MRC: Medical Research Council; PAID: Problem Areas in Diabetes questionnaire; MID: Minimal important differences; SD: Standard deviation.

## Competing interests

The authors declare that they have no competing interests.

## Authors’ contributions

LdV, GN, EvtR and AvdH are responsible for the design of the study with comments of CB, GR and MR. PK provided advice on the statistical analysis plan. All authors contributed to revising the article. All authors read and approved the final manuscript.

## Pre-publication history

The pre-publication history for this paper can be accessed here:

http://www.biomedcentral.com/1472-6823/14/21/prepub
